# Supplementary material for: An African origin for Mycobacterium bovis
Source: Evol Med Public Health. 2020 Jan 31;2020(1):49–59. doi: 10.1093/emph/eoaa005 (PMC7081938; doi:10.1093/emph/eoaa005)
Supplement: eoaa005_Supplementary_Data [file eoaa005_supplementary_data.zip › Loiseau_et_al_SupplementaryMaterial_REVISION.docx]

**Supplementary Figures:**

**Figure S1** – Flow chart showing the selection of genomes.

**Figure S2** - Geographic distribution of the *M. bovis* samples with unknown classification used in this study according to isolation country.

**Figure S3** – Maximum likelihood phylogeny of all 3364 genomes, based on 45 981 variable positions. The scale bar indicates the number of substitutions per polymorphic site. The phylogeny is rooted on a *M. tuberculosis* Lineage 6 genome from Ghana. The outer ring indicates the geographical region from which the strains were isolated. The four clonal complexes are highlighted on the tree. Branches corresponding to BCG genomes are coloured in grey and the *PncA* mutation H57D is indicated by a yellow star.

**Figure S4** – Phylogeographic reconstruction of *M. bovis* and *M. caprae*, inferred from 392 genomes. Thirteen UN-defined geographic regions were assigned to the discrete character geographic origin, and mapped onto the phylogeny. Pie charts at internal nodes represent the summary posterior probabilities (from 100 runs) of the reconstructed ancestral geographic states and are coloured according to geographical UN region**.**

**Figure S5** – A) Tip-to-root regression and B) Date randomization tests (DTR). The confidence interval of the clock rate estimate for the observed data does not overlap with the confidence intervals of the clock rate estimates obtained from the randomized sets.

**Supplemental Tables:**

**Table S1 -** List of genomes included in this study along with metadata used for the analyses.

**Table S2 -** Comparison of models for discrete character evolution using likelihood ratio tests.

**Table S3 –** Results of all BEAST analyses.

**Table S4 -** Spoligotype patterns determined *in silico* for different clonal complex groups with reference to other studies.

**Supplementary files:**

**TreeS1-S10** Ten time-calibrated trees resulted from the molecular clock analyses. The file names indicate the software used (LSD or BEAST), the subsample, and the coalescent population prior (BSP: Bayesian Skyline; exponential: exponential population growth; constant: constant population size).  Tip labels are present in Table S1. Ages in years before present can be visualized as well as the 95% High Posterior Density (HPD) (BEAST trees) using FigTree [72].

[72] Rambaut A; FigTree. Edinburgh: Institute of Evolutionary Biology, University of Edinburgh, 2010.
