# Supplementary figures and images for: An African origin for Mycobacterium bovis
Source: Evol Med Public Health. 2020 Jan 31;2020(1):49–59. doi: 10.1093/emph/eoaa005 (PMC7081938; doi:10.1093/emph/eoaa005)

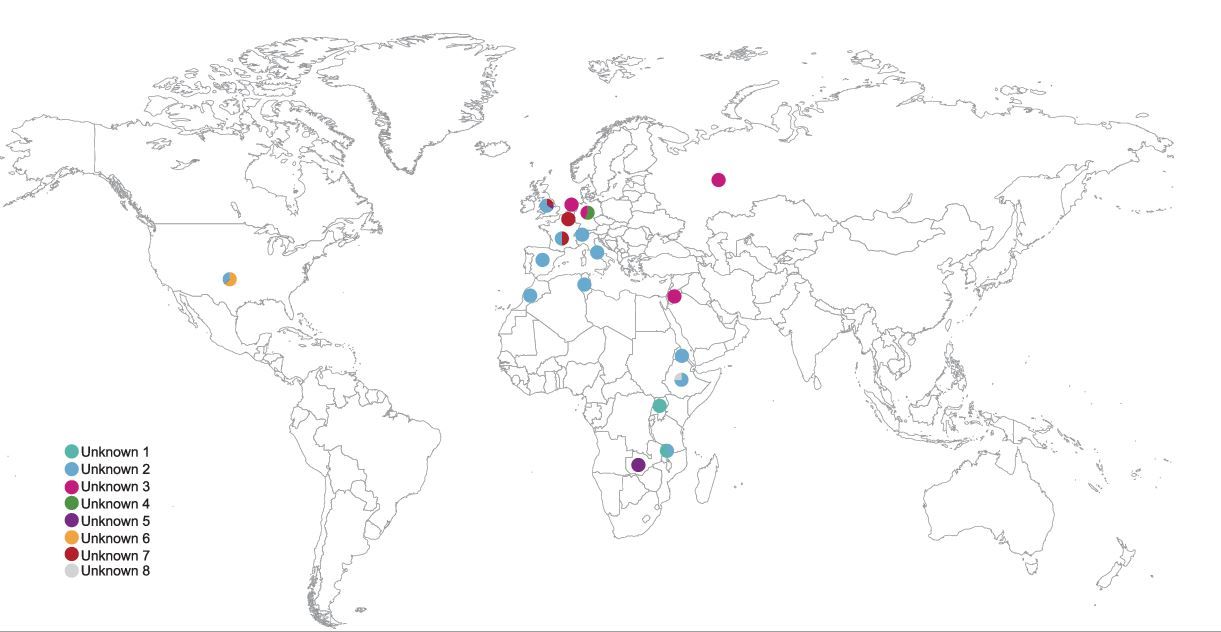

Supplement: eoaa005_Supplementary_Data [file eoaa005_supplementary_data.zip › Fig S2.JPG]

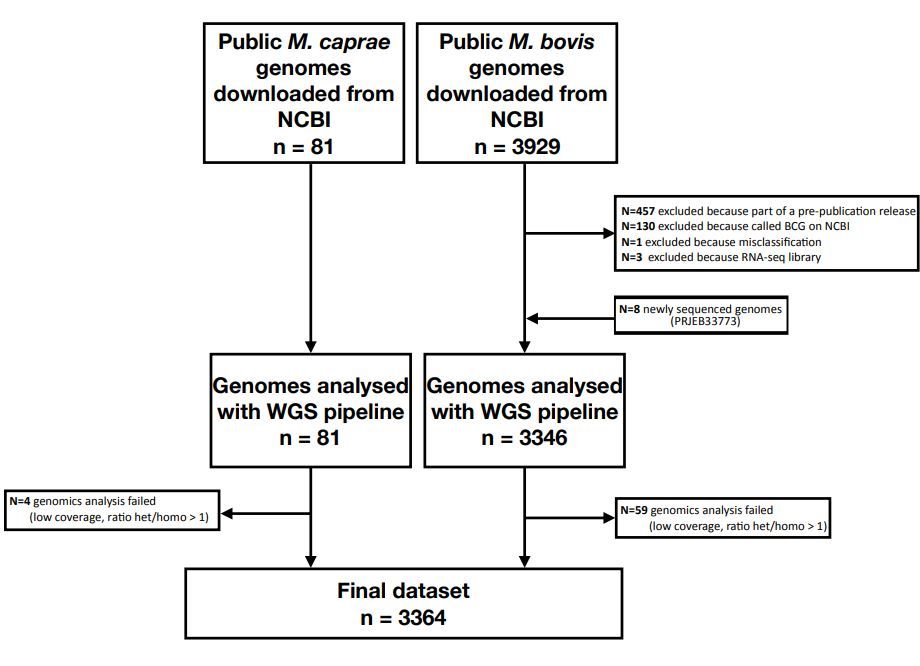

Supplement: eoaa005_Supplementary_Data [file eoaa005_supplementary_data.zip › Figure S1.JPG]

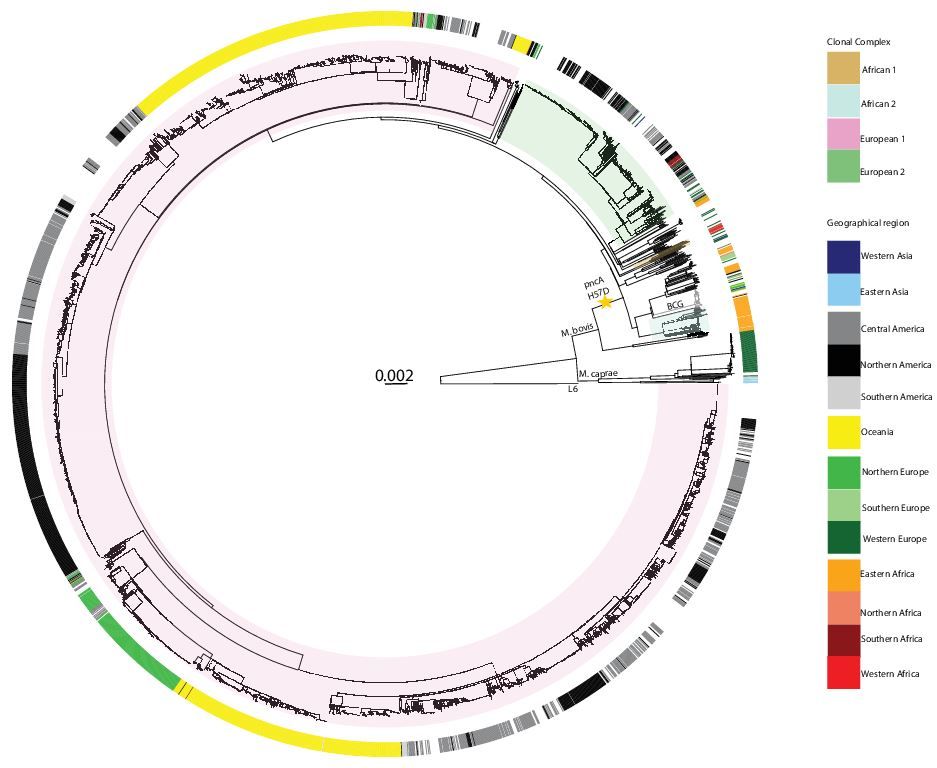

Supplement: eoaa005_Supplementary_Data [file eoaa005_supplementary_data.zip › Figure S3.JPG]

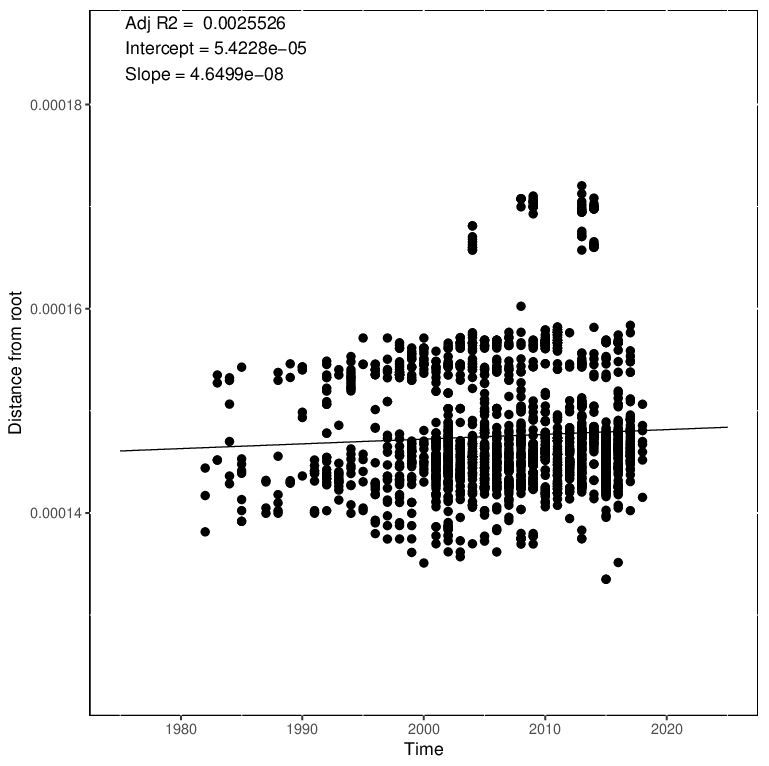

Supplement: eoaa005_Supplementary_Data [file eoaa005_supplementary_data.zip › Figure S5A.JPG]

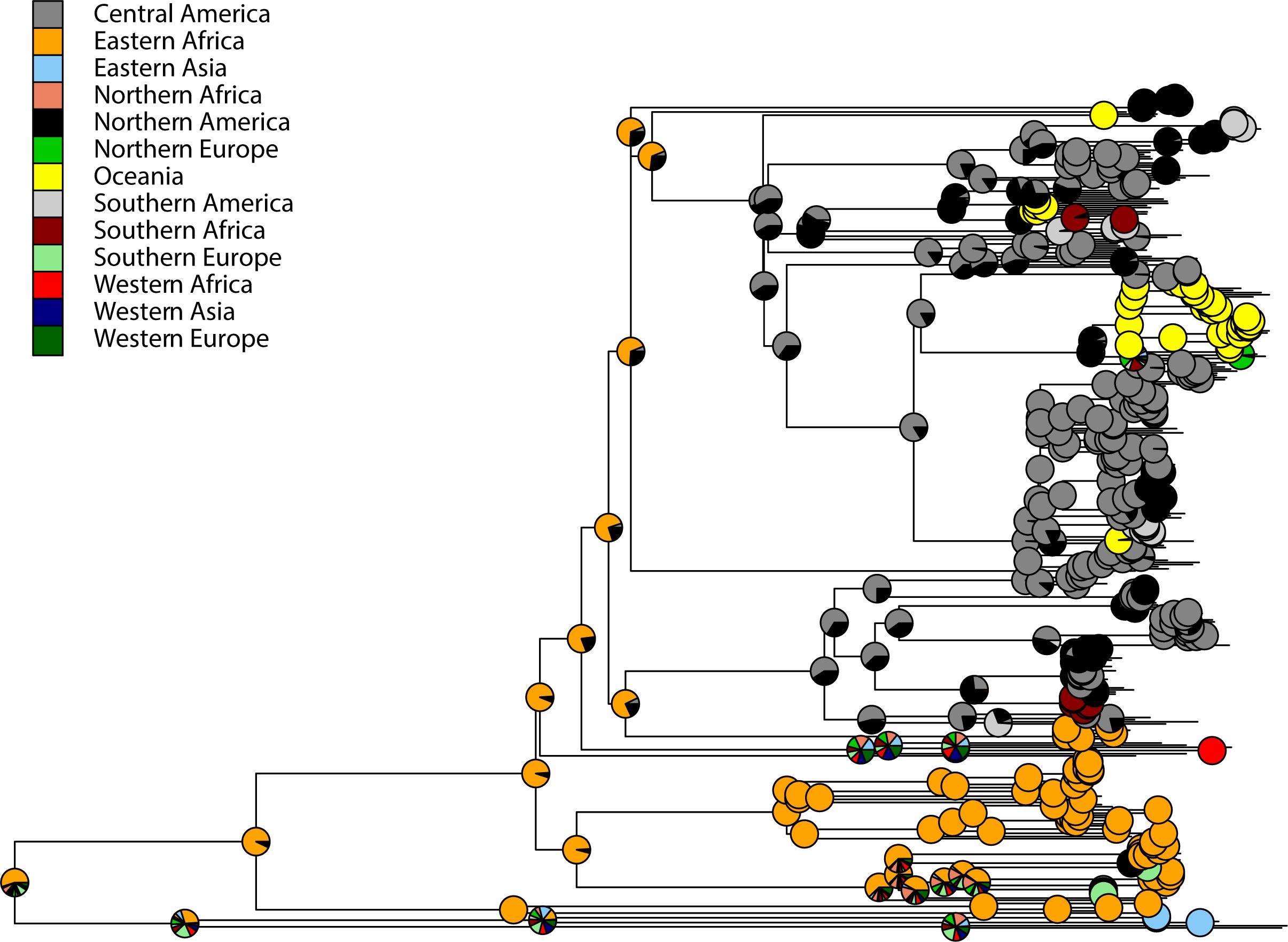

Supplement: eoaa005_Supplementary_Data [file eoaa005_supplementary_data.zip › FigureS4_new.jpg]

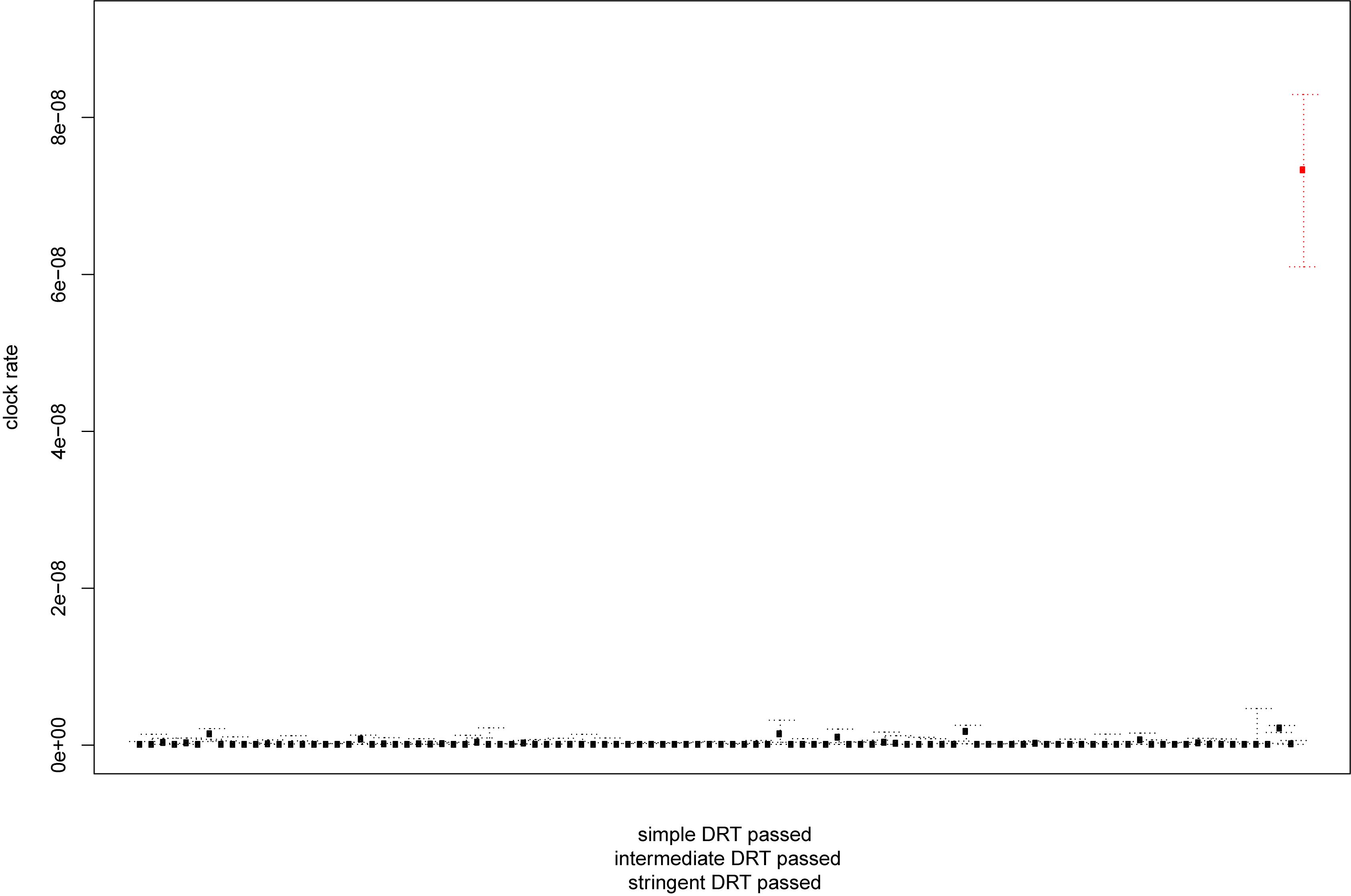

Supplement: eoaa005_Supplementary_Data [file eoaa005_supplementary_data.zip › FigureS5_B.jpg]
