# Supplementary material for: An African origin for Mycobacterium bovis
Source: Evol Med Public Health. 2020 Jan 31;2020(1):49–59. doi: 10.1093/emph/eoaa005 (PMC7081938; doi:10.1093/emph/eoaa005)
Supplement: eoaa005_Supplementary_Data [file eoaa005_supplementary_data.zip › TableS2.doc]

Table S2 – Model selection of the best fitting model describing the transitions between different geographic origins for M. bovis and M. caprae. ** result of fitMK function *one-sided Chi-square distribution.

| Models | Likelihood Ratio Test** | Number of parameters estimated | p-value* |
| --- | --- | --- | --- |
| *ER vs SYM* | LTR=151.2505 | 1 | 0.0000009 |
| *ER vs ADR* | LTR= 182.14 | 78 | 0.06 |
| *SYM vs ARD* | LTR= 30.89 | 156 | 0.9 |
